# 

**Published:** 1889-03-16

**Authors:** 


### CONTENTS.

**Doctors' Disabilities** - 371

**Stirpiculture** - 372

**The Doctor's Pulpit.**—Man and His Enemies - 373

**Police Matrons** - 374

**Turning Over New Leaves** - 374

**Military Graveyards and Epitaphs in India**—III. By an OLD INDIAN - 375

**In a Coal Mine with Miners.**—II. By a NEW HAND - 377

**Notes and News** - 378

**Everybody's Page** - 380

**Annotations.**—From the Court to the Hospital.—The Brain as a Working Tool.—"Common Humanity" in Lancashire - 381

**Glances at the Insane in Other Lands**—VII.—Alabama - 382

**Bath and Her Waters** - 382

**False Impressions.** By CAROLINE FOTHERGILL, Author of "An Enthusiast," "A Voice in the Wilderness," etc. - 383

**Hints for the Sick Room.** By M. M. BASIL, M.A., M.B., etc. - 385

**Scraps and Gleanings** - 386

**Amusements and Relaxation** - 386

### EXTRA SUPPLEMENT—THE NURSING MIRROR.

**En Passant.**—National Pension Fund for Nurses.—Scotch Nursing.—Nurse and Watcher.—Pension for Reading Nurses.—Ladies as Nurses - xciii
**Nursing Sisterhoods.**—The Community of St. John the Divine - xciv
**Religious or Lay Nursing?**—The Question in Paris - xciv
**Work at Durham** - xcv
**Nursing the Insane** - xcv
**Everybody's Opinion.**—A Nurse's Savings Fund - xcvi
**Appointments, Vacancies, Notes and Queries** - xcvi

